# Mongolian Child’s Play and the Gut Microbiome

**DOI:** 10.4269/ajtmh.18-0596

**Published:** 2018-12

**Authors:** Hilary Ong

**Affiliations:** Department of Pediatrics, Children’s Hospital Los Angeles, Los Angeles, California

Three hours into our rickety ride in a four-wheel drive Jeep, the charm and beauty of the Gobi Desert was wearing off. There was no human in sight, but periodic herds of Mongolian buffalos, horses, camels, and sheep appeared in the horizon to provide me with a burst of energy. My coresident and I had traveled 30-plus hours from Los Angeles to the Gobi to visit a Mongolian nomadic herding family. This was my first visit to the vast summer grasslands of the Gobi, but my third visit to Mongolia for my global health research project on environmental influences on child health as a pediatric resident.

When our Jeep finally came to a halt, I sprung out at the first opportunity to stretch my arms until I could reach the clear, blue sky. After a few moments to myself, our hosts invited us into their Ger (a traditional Mongolian tent house). As we sat drinking bowls of unpasteurized goat’s milk (yes, unpasteurized) and gnawing on rock hard *hurood* cheese curds, our host family abruptly stood up and walked outside.

It was nice to emerge from the Ger into the gentle warm sunlight. We saw Grandmother, a 5-foot-tall woman wearing a colorful headscarf exposing her hard-won, sun-tested wrinkles, herding her goats close to the camp. It was milking time. Without prompting or coercing, the goats magically aligned in a row. With craft and efficiency, Grandmother grabbed the goats by their horns and interlocked the horns alternating sides. She then secured this braid of goats with a thick rope of animal hair by intertwining the rope around the goats’ neck. Just like that, without complaint, 40 goats with their full utters were neatly lined up to be milked.

I gestured to Grandmother to let me have a go at milking. She smiled and handed me a bucket. I was elated. As I eagerly approached the goat braid, still amazed at the efficiency and craftsmanship with which Grandmother had organized them, I soon realized a problem: I had never milked an animal before.

Two goats later, my pail was a 10th full, meanwhile Grandmother had milked a third of the herd. My back hurt and thighs burned from squatting, but Grandmother was going strong. Yes, I admit, I have the strength and stamina of a city girl. I proudly brought my pail to Grandmother and we both had a good laugh. Grandmother gestured for me to taste the fresh goat’s milk. I looked down into the pail of milk with bits of hair and dirt floating atop. Picturing my infectious disease attendings, a variety of disease-causing bacteria found in raw milk raced through my mind—*Brucella*, *Listeria*, *Campylobacter*, *Cryptosporidium*, *Escherichia coli*, and *Salmonella*. I was hesitant to refuse Grandmother’s kindness, but I did not think my immune system and intestinal microbiome could handle even a small sip. My American city girl microbiome is not the same as the unique composition and function of Mongolians’ gut microbiotia.

As I learn more about how the gut microbiome protects against infectious diseases, I cannot help but wonder whether Grandmother has a more hardy gut flora and immune system than I do. I knew from my studies that Mongolians who live in pasturing areas, compared with those who live in an urban city, have higher amounts of *Lactobacillus* and *Bifidobacterium* and lower amounts of Enterobacterium in their gut microbiota. Living environments play a major role in microbiota composition and functionality. I remembered reading an animal study that found an increase in gut microbial diversity when the adult mice lived in close proximity to goats. *Yes, indeed. Grandmother is more hardy than I am.*

In the end, after much debate in my mind, I respectfully declined Grandmother’s kind offer. I snapped a few iPhone-quality picturesque photographs of the goat braid and moved on to play with the children. The two boys, ages 5 and 8 years, were chasing around baby goats. With much difficulty, I finally captured a baby goat and snuggled it for a few moments before it jumped out of my arms. Capture and release, capture and release; we ran around and lost track of time.

To the side of our child’s play, I saw Grandmother placing a 10-pound bag of white sugar next to a big pail of goat’s milk. The children dropped their baby goats and ran toward the bag of sugar. Their pudgy dirt and goat-hair-laden hands excitedly reached to grab handfuls of sugar. One fist full after another, the children’s smiles were soon encircled with sticky white specks. *Should I intervene if the children were to get past half the bag?* My worries were laid to rest when the children’s attention suddenly snapped backed to the goats as one stray baby goat timidly approached them. The 8-year-old was persistent in chasing and teasing this little goat. Minutes later, he was far into the horizon.

His younger brother, on the other hand, puttered out and plopped himself onto a patch of grass just a few feet away from me. He was carefully inspecting his hands and arms for patches of sugar and happily licked off the remaining sweet treat. When that was all and done, he found a new distraction. Surrounding him were cakes of dried animal dung. He picked up one cake and crushed it to pieces, then another cake, and another one, until he was able to build a small hill of crushed dung pieces. He then sunk his little hands into the pile, pulled out two big handfuls, and ate them. My eyes widened in disbelief. He turned toward me to give me a mischievous smile.

The pediatrician and public health scientist in me kicked in. Pastoral Mongolians’ lifestyle, living environments, and dietary habits rich in fermented dairy products such as *hurood* cheese all contributed to their unique gut microbiome exposome. Mongolians have an enriched gut microbiota with *Lactobacillus* and *Bifidobacterium*, both considered probiotics with beneficial health effects such as inhibition of pathogenic intestinal organisms and regulation of local and systemic immune responses. These probiotics decrease the duration of acute infectious diarrhea in infants and children. *Does periodically eating fistfuls of animal feces for fun protect against infections? Would the young boy get infectious diarrhea? Or was he “immune” to his environment? What effect does the consumption of animal feces, unpasteurized hurood cheese, and goat's milk have on children’s gut microbiome?* An avalanche of questions raced through my mind.

With the backdrop of the Mongolian setting sun, I contemplated the relevance of these questions and considered the necessity of multidisciplinary approaches to the study of the microbiota in relation to infectious diseases, specifically in traditional communities. A day in the life of a Mongolian nomadic herder showed me how health is closely intertwined with the genetic, biological, social, and ecological environment. For, in rural Mongolia, humans, animals, and nature appeared to live in perfect harmony. As my mind filled with ideas of future global health collaborations, our Jeep drove away from the Ger tent. I watched as one by one—the boys, goats, Grandmother, and Ger—became engulfed by the surrounding grassland.

